# Study protocol for a multicentre randomized controlled trial on effectiveness of an outpatient multimodal rehabilitation program for adolescents with chronic musculoskeletal pain (2B Active)

**DOI:** 10.1186/s12891-016-1178-5

**Published:** 2016-07-28

**Authors:** Carolien Dekker, Mariëlle E. J. B. Goossens, Caroline H. G. Bastiaenen, Jeanine A. M. C. F. Verbunt

**Affiliations:** 1 Department of Rehabilitation Medicine, CAPHRI, Program Functioning and Rehabilitation, Maastricht University, Postbus 616, 6200 MD Maastricht, The Netherlands; 2 Department of Clinical Psychological Sciences, EPP, Maastricht University, Maastricht, The Netherlands; 3 Department of Epidemiology, CAPHRI, Program Functioning and Rehabilitation, Maastricht University, Maastricht, The Netherlands; 4 Maastricht University Medical Centre, Maastricht, The Netherlands; 5 Adelante Centre of Expertise in Rehabilitation and Audiology, Hoensbroek, The Netherlands

**Keywords:** Chronic pain, adolescent, Graded Exposure Therapy, RCT, Pragmatic, Economic evaluation, Rehabilitation

## Abstract

**Background:**

Chronic musculoskeletal pain (CMP) in adolescents can influence functioning and well-being, and has negative consequences for families and society as well. According to the Fear Avoidance Model, fear of movement and pain catastrophizing can influence the occurrence and maintenance of chronic pain complaints and functional disability. Primary objective is to evaluate the effectiveness of a multimodal rehabilitation program in reducing functional disability for adolescents with CMP compared with care as usual.

**Methods/Design:**

Pragmatic multicentre parallel group randomized controlled trial. Randomization by minimization (ratio 1:1) and treatment allocation will be concealed, computer-generated and performed by an independent organization. After randomization, data collection and researchers remain blinded. Inclusion of 124 adolescents and their parents is intended. This sample size is based on a 25 % difference in group mean on the primary outcome, with α = 5 %, β = 80 % and expected 15 % loss to follow up. Study population are adolescents (12–21 years) with CMP with an indication for outpatient rehabilitation treatment in the Netherlands.

The intervention group receives a Multimodal Rehabilitation Program (MRP), a multidisciplinary outpatient individual rehabilitation program. MRP consists of 2 different treatment approaches: A graded exposure module or a combination module of graded exposure and physical training. Selection of a module depends on the needs of the patient. To both modules a parent module is added. The control group receives care as usual, which is the care currently provided in Dutch rehabilitation centres. Treatment duration varies between 7 and 16 weeks, depending on treatment allocation.

Self-reported measurements are at baseline, and at 2, 4, 10 and 12 months after start of treatment. Intention to treat analysis for between group differences on all outcome variables will be performed. Primary outcome is functional disability (Functional Disability Inventory). Secondary outcome variables are fear of pain, catastrophizing, perceived harmfulness, pain intensity, depressive symptoms, and quality of life. Total direct and indirect costs and health related quality of life will be measured. Process evaluation focuses on protocol adherence, patient centeredness and treatment expectations.

**Discussion:**

A pragmatic approach was chosen, to ensure that results obtained are most applicable to daily practice.

**Trial registration:**

Clinicaltrials.gov ID: NCT02181725 (7 February 2014).

Funded by Fonds Nuts Ohra, Stichting Vooruit, and Adelante.

## Background

Chronic pain in children and adolescents is a major health concern in the paediatric population [1–3]. Up to 25 % of Dutch schoolchildren, especially adolescents, report pain for 3 months or longer (=chronic pain) [1, 4]. Internationally, prevalence rates for musculoskeletal pain vary between 11 and 38 % [3]. Chronic musculoskeletal pain is, together with headache and abdominal pain, one of the most reported pain complaints in adolescents [1, 5]. Although a broad array of medical diagnoses is involved in adolescent pain conditions, in only 10–30 % of cases a specific medical disease explaining the pain is identified [6, 7]. Unexplained musculoskeletal pain is not self-limiting: persistence rates of pain up to 30–64 % after 4 years have been reported [5, 8, 9]. Pain during adolescence even increases the risk of having a chronic pain syndrome in adulthood [5, 10].

### The consequences of musculoskeletal pain

Most adolescents function quite well regardless of pain. Nonetheless, in approximately 40 % of the adolescents with chronic pain, pain has a disabling impact on daily functioning [11–13]. Pain can interfere with developmental, school and leisure time activities, and causes serious psychological distress. It also has an impact on the adolescent’s families. Primary caregivers report more restrictions in social life and more problems with coping with the adolescent’s pain [14–16]. In addition to the psychological burden, there is little known about the financial burden of caring for a child with chronic pain [17–19].

Although the body of evidence on adult chronic pain and different rehabilitation treatments is growing, research into adolescents who suffer from chronic pain complaints is still in its infancy. Rehabilitation care for adolescents with chronic pain differs from adult care in several ways. First, in adolescent chronic pain rehabilitation, the family system, and especially influences of the parents are important to take into account. Parental behaviour, such as protective behaviour, has been shown to influence adolescent’s response to pain. This can result in greater functional disability, higher school absence and more depressive symptoms in the children [15, 20–25]. Second, situations and activities that contribute to developing and maintaining adolescent chronic pain are not completely similar to those contributing to adult chronic pain [26, 27]. Third, in adolescents with chronic pain, prevalence rates of hypermobility (40–55 %) are higher than in healthy adolescents. Hypermobility as such is not a problem, but when hypermobility is associated with complaints, such as pain, it is called joint hypermobility syndrome (HMS) [28, 29] thereby making HMS a contributing factor in treating adolescent chronic pain [30–33].

### Theoretical framework

According to the fear avoidance model of chronic pain [34], both fear of pain/movement and catastrophic thinking about pain can lead to the development and maintenance of chronic pain problems. Simons and Kaczynski [35] studied the applicability of the fear avoidance model in the paediatric population and concluded that the model is applicable, after making some modifications to account for developmental aspects at child/adolescent age. Goubert and Simons [25] presented an interpersonal fear avoidance model of pain, in which the social context of the adolescent becomes clear.

The implications of these models for a treatment approach of adolescent CMP is that lowering fear of pain/movement and accompanying catastrophic thinking could lower avoidance behaviour and increase functional ability [34]. Moreover, the interpersonal fear avoidance model accentuates the necessity to involve the family system as part of the treatment, in a way that a context can be created in which the adolescent can change. Adding the parental influences to the fear avoidance model is an important difference between the model for adults and the model for adolescents.

### Treatment of chronic pain

Adolescent chronic pain is regarded a complex health problem that requires a multidisciplinary treatment approach from a biopsychosocial perspective [17, 36, 37]. This study focuses on multidisciplinary outpatient treatment for adolescents with CMP. Outpatient treatment currently provided to adolescents in the Netherlands is mostly based on the principles of Graded Activity (GA). There is a need for more RCT’s that investigate effectiveness of different patient (and parent) interventions in paediatric CMP [17, 22, 38]. The MRP that is investigated in this study is based on Graded Exposure therapy (GE). GE specifically aims at challenging catastrophizing thoughts and performing feared activities in order to improve functional ability of patients with chronic pain. In adults with chronic pain this treatment approach is successful [39, 40]. The Maastricht University Medical Centre (MUMC) has recently developed a multimodal rehabilitation program (MRP), aimed specifically at improving adolescent’s functional ability by reducing pain-related fear. The Multimodal Rehabilitation Program consists of 2 different treatment approaches: A graded exposure module or a combination module of graded exposure and physical training. Both approaches also include a parent module. The choice of graded exposure module or combination module depends on the needs of the patient (Table 1).

**Table 1:** Intervention group: Multimodal Rehabilitation Program, description of the treatment

Graded Exposure module	OR	Combination module (Graded exposure and physical training)
*Phase 1 Intake and PHODA-Youth (60 min)* • Information about cognitive, behavioral and psychophysiological aspects of pain complaints • Description of family system and situation • Identification of activities that are being avoided, and accompanying cognitions *Phase 2 Education (60 min)* • Explanation of treatment rationale • Explanation of treatment • Alternative explanation for persistence of pain complaints • Personal fear avoidance model *Phase 3 Graded Exposure treatment sessions (12 × 60 min)* • Systematic exposure to fear provoking activities and movements • Behavioral experiments • Generalization and relapse prevention		*Phase 1 Intake, PHODA-Youth, and physical examination (60 min)* • Information about cognitive, behavioral and psychophysiological aspects of pain complaints • Description of family system and situation • Identification of activities that are being avoided, and accompanying cognitions • Physical examination of strength, balance and propriocepsis. *Phase 2 Education Graded Exposure and training (60 min)* • Explanation of treatment rationale • Explanation of treatment: Training and graded exposure sessions • Alternative explanation for persistence of pain problems • Explanation of physical training • Personal fear avoidance model *Phase 3 Training (16x120 min)* • Training (hydrotherapy if possible), focusing on aerobic capacity, muscle strength, core stability, propriocepsis • Homework (training exercises) *Phase 4 Graded Exposure treatment sessions (6x 60 min)* • If necessary: repetition of treatment rationale • Systematic exposure to fear provoking activities and movements • Behavioral experiments • Generalization and relapse prevention
	AND	
Parent module *Phase 1 Medical education and treatment rationale* • Explanation of treatment rationale • Explanation of treatment • Alternative explanation for persistence of pain complaints • Personal fear avoidance model for their child *Phase 2 The role of pain within the family system* • Explanation of the influence of pain on the family system and interaction between parents and child • Tools to support the child in creating and maintaining behavioral change *Phase 3 Generalization and relapse prevention* • Identifying and recognizing risky situations that can trigger relapse • Preventing relapse by making plans		

### Aims of the study

This study investigates the effectiveness of a multimodal rehabilitation program (MRP) including Graded Exposure treatment in reducing functional disability (measured with the Functional Disability Inventory on short and long term) in adolescents (12–21 years) with chronic musculoskeletal pain compared with care as usual (CAU).

Secondary aims are *a)* to compare the cost-effectiveness and cost-utility of both interventions, also during the follow-up period, an *b)* to evaluate the feasibility of working with MRP in terms of treatment fidelity and patient centeredness.

## Methods

### Study design and participants

A two group pragmatic randomized controlled trial is undertaken with adolescents with CMP, with follow-up assessment at 2, 4, 10 and 12 months after start of treatment. Adolescents will be allocated to either intervention (MRP) or control (CAU) with a ratio 1:1. Patients who receive an indication for outpatient rehabilitation treatment will be invited to participate and will be randomized to either usual care (CAU) or the intervention (MRP) after informed consent is obtained. Parents will be asked to participate as well.

Estimates for the calculation of the sample size are based on the primary outcome measure, the Functional Disability Inventory (FDI). An average FDI total score of 23 (average score obtained from adolescents that were treated at the Maastricht University Medical Centre in the year before the start of this study), a standard deviation of 9.2 and an expected mean difference between intervention and control condition of 5 points on the total FDI-score were used. A difference of 5 points equals approximately a 25 % difference in mean FDI scores between intervention and control group.

Given α = 0.05, two sided testing, a power of 80 %, and anticipating 15 % loss to follow-up, a sample size of 62 participants per trial-arm was calculated. For two trial arms, this results in a total sample size of *N* = 124.

Adolescents will be recruited from 4 centres in The Netherlands: Maastricht University Medical Centre/Adelante, Laurentius Hospital Roermond, Revant Rehabilitation centre Breda and Rijndam Rehabilitation Centre in Rotterdam. Both treatments offered are embedded in the daily care process in each treatment centre, with an inclusion period of 1.5 years. Ethical approval for this trial was granted by the Medical Ethics Committee Academic Hospital Maastricht/Maastricht University, the Netherlands, NL47323.068.13/METC13-3-062.

### Eligibility criteria

Adolescents with a treatment indication for outpatient rehabilitation care for treatment of CMP, aged between 12–21 years and with adequate Dutch literacy were eligible for inclusion. Patients will be excluded if there is a) any suspicion of a medical (orthopaedic, rheumatic or neurological) disease, that can fully explain the current level of severity of pain complaints, b) any suspicion of an (underlying) psychiatric disease that hampers rehabilitation treatment or c) pregnancy.

### Randomization and allocation concealment

Patients will be randomized by minimization after informed consent is obtained. Minimization [41] was chosen to balance treatment centre, sex and age factors within the study. To execute the randomization procedures a validated electronic randomization system (ALEA, offered by the Clinical Trial Centre Maastricht, CTCM) will be used. Randomization and treatment allocation are completely independent of the study and blinded for participants including parents and all researchers, including raters and at that moment for caregivers and the consultant in rehabilitation medicine. After treatment allocation is revealed, blinding cannot be maintained for care givers. Adolescents and parents will be kept naïve concerning the comparison between the interventions. The research team remains blinded during the course of the study. Data collection is also blinded.

### Interventions

Contrast between the interventions is found in their theoretical basis and point of engagement where the treatment focuses on. Graded Exposure uses principles from classical conditioning and cognitive therapeutic techniques to systematically reduce pain-related fear and catastrophic thinking in adolescents with chronic pain [42]. Graded Activity uses principles of operant conditioning, resulting in rewarding healthy and age adequate daily functional activities performed by the adolescent and a stepwise increase of the adolescent’s activity levels [42].

Parents are present and are invited to participate in both the intervention and control condition. However, in the intervention condition (MRP), parents receive additional 3 group meetings (the parent module) with the treatment team (Table 1). Meaning that in the MRP parents are involved in the treatment of their adolescent in two ways. They are both present and participate during treatment of their child, but additionally they participate in a separate parent module.

Both treatments are provided by a multidisciplinary treatment team, including a consultant in rehabilitation medicine, a physiotherapist or occupational therapist and a psychologist/behavioural therapist.

#### Intervention group

In Table 1 the elements of the three modules of the MRP are described. Graded Exposure aims to increase healthy behaviour by systematically and gradually exposing adolescents to fear provoking activities and movements. Starting point for this approach is a personal hierarchy of activities feared by the adolescent. The Photograph series of Daily Activities – Youth (PHODA-Youth) [27] is used as a standardized procedure to build these personal hierarchies. All therapists in the MRP condition received training on the principles of Graded Exposure therapy and the treatment protocols, consisting of a workshop and a 3-day training provided by skilled trainers (with 3 to >15 year experience in Graded Exposure therapy). Administration of the PHODA-Youth was part of the 3-day training.

Adolescents in the intervention condition MRP receive a graded exposure module (7 weeks) or a combination module (15 weeks) with training and graded exposure. Their parents receive a parent module (3 meetings). Adolescents with CMP receive the graded exposure module. If an adolescent is additionally identified (Brighton criteria including Beighton score, will be used [29, 32, 43]) as having pain complaints related to joint hypermobility (HMS), the combination module will be offered. All parents in the MRP condition are offered the parent module, which is organised in the evening hours.

#### Control group

Currently, treatment of adolescents with chronic pain in a rehabilitation setting consists of multidisciplinary treatment based on the principles of Graded Activity. Table 2 presents the main treatment elements of GA. GA aims to increase healthy behaviour by employing operant learning principles such as encouraging desired behaviours [42]. The treatment consists of a time contingent and stepwise increase in the level of functioning, despite complaints of pain [44]. Duration of the care as usual treatment varies between 9 and 16 weeks. This variation exists because each collaborating treatment centre has developed its own specific treatment protocol, also depending on the practical and logistic possibilities of the treatment centre. Content of the treatment programmes is generally similar and principal discriminative from the content of the experimental intervention (MRP).

**Table 2:** Control group: Care as Usual, description of the treatment

*Phase 1 Inventory of the problem* • Anamnesis • Estimation of motivation for rehabilitation treatment • Physical examination • Identification of maintaining factors for the pain problems • Analysis of activities *Phase 2 Problem analyses* • Ordering information from inventory • Providing an explanatory model for the pain problem • Treatment plan *Phase 3 Education* • Exploring willingness to change behaviour • Changing behaviour despite pain *Phase 4 Choosing activities* • Choosing activities on a functional level • Determining treatment goals (SMART) *Phase 5 Determining baseline (Pain contingent functioning)* • Determining baseline level of functional activities (min 3–5 measurement moments) *Phase 6 Determining goal and scheme to increase activity* • Starting level below baseline • Scheme for time contingent increase in activities • Establishing treatment goals *Phase 7 Executing scheme* • Time contingent increase of activities • Encouragement of successful behaviour • Generalization at home and school *Phase 8 Generalization and evaluation* • Applying in daily situations • Evaluation of treatment	

### Outcome measures

PedIMMPACT recommendations were followed when choosing outcome domains and in the selection of measurement instruments for this study [45]. Details of measurement instruments can be found in Table 3. Assessments will be performed at baseline (before start of treatment) and at 2, 4, 10 and 12 months after start of treatment. Primary outcome measure is adolescent’s functional disability (FDI) [46, 47]. Secondary outcome measures are adolescent fear of pain (FOPQ-C) [48], perceived harmfulness (PHODA-Youth) [27], pain catastrophizing (PCS-C) [49, 50], pain intensity (VAS) [51], depressive symptoms (CDI) [52] and pain specific quality of life (QLA-CP) [53], and parent perceived functional disability (FDI-P) [46, 47], fear of pain (FOPQ-P) [48], pain catastrophizing (PCS-P) [50], adult responses to children’s symptoms (ARCS) [54].

**Table 3:** Primary and secondary outcome measures^a^

Primary outcome	Measurement instrument	Time points of measurement
Functional disability [46, 47]	Functional Disability Inventory (FDI)	Baseline, 8 weeks, 16 weeks, 10 and 12 months
	Self-report measure for perceived difficulty in performing activities at school, at home and in recreational or social interactions.	
	15 Items rated on a five-point scale (0–4). Total scores range 0–60 with a higher scores indicating greater disability.	
	Valid and reliable measure for assessing pain related disability in adolescents.	
ᅟ	ᅟ	ᅟ
Secondary outcomes	Measurement instrument	Time points of measurement
Fear of pain [48]	Fear of Pain Questionnaire (FOPQ-C)	Baseline, 8 weeks, 16 weeks, 10 and 12 months
	Self-report measure to assess pain-related fear in adolescents with chronic pain. 24 items rated on a five-point scale (0–4). Total scores range 0–96 with a higher score indicating more fear. Consists of subscales 1) Fear of Pain and 2) Avoidance of activities.	
	Psychometric properties of the English version are good.	
Perceived harmfulness [27]	PHODA-Youth	Baseline, 8 weeks, 16 weeks, 10 and 12 months
	Measures perceived harmfulness of physical and social activities and can be used to create a hierarchy of fearful activities.	
	51 photographs are rated on a scale 0–10 (steps 0.1). The higher a photo is ranked, the more harmful the adolescent thinks executing the activity is. Consists of subscales 1) activities of daily living, 2) intensive physical activities and 3) social activities.	
	Psychometric qualities are good.	
Pain Catastrophizing [49]	Pain Catastrophizing Scale (PCS-C)	Baseline, 8 weeks, 16 weeks, 10 and 12 months
	Self-report measure of catastrophic thinking about pain. Frequency of feelings and thoughts adolescents may experience when they are in pain are measured.	
	13 items rated on a five-point scale (0–4). Total scores range 0–52 with higher scores indicating more catastrophic thinking. Consists of subscales 1) Rumination, 2) Magnification, and 3) Helplessness. Reliability and validity are good.	
Depressive symptoms [52]	Child Depression Inventory (CDI)	Baseline, 8 weeks, 16 weeks, 10 and 12 months
	Self-report measure of depressive symptoms in children and adolescents.	
	27 items rated on a three-point scale (0–2). Total scores range 0–54 with higher scores indicating more depressive symptoms. Consists of subscales 1) Negative mood, 2) Interpersonal problems, 3) Ineffectiveness, 4) Negative self-esteem, and 5) Anhedonia.	
	Dutch version demonstrates good reliability and validity.	
Pain specific quality of life [53]	Quality of Life in Adolescent with Chronic Pain (QLA-CP)	Baseline, 8 weeks, 16 weeks, 10 and 12 months
	Self-reported pain-specific quality of life measure for adolescents with chronic pain.	
	44 items in 6 domains, 1) Psychological functioning, 2) Functional status, 3) Physical status, 4) Social functioning, 5) Satisfaction with life in general, and 6) Satisfaction with health. A high score on each domain of the questionnaire represents a better quality of life.	
	Internal consistency and construct validity have shown to be adequate.	
Pain Intensity [51]	Visual Analogue scale (VAS)	Baseline, 8 weeks, 16 weeks, 10 and 12 months
	Self-report measure of pain intensity. Sliding scale where the ends of the line represent the extreme limits of pain intensity (no pain at all and worst pain imaginable). Average VAS score is taken of pain at this moment, worst and least pain in last week.	
	Reliable method in children above 8 years old. Sound psychometric properties and clinical utility.	
Parent perceived Functional Disability [46, 47]	Functional Disability Inventory – Parent report (FDI)	Baseline, 8 weeks, 16 weeks, 10 and 12 months
	Measure of parent perceived functional disability of their child.	
	15 items rated on a five-point scale (0–4). Total scores range 0–60 with higher scores indicating greater parent-perceived difficulty in performing activities.	
Parent perceived Fear of Pain [48]	Fear of Pain Questionnaire – Parent report (FOPQ-P)	Baseline, 8 weeks, 16 weeks, 10 and 12 months
	Parent proxy report measure to assess parents perception of their childs pain-related fear experience.	
	23 items rated on a five-point scale (0–4). Total scores range 0–92 with higher scores indicating a higher parent perceived fear. Consists of subscales 1) fear of pain, 2) avoidance of activities, and 3) School avoidance.	
	Psychometrically sound measure with strong internal consistency, good construct and criterion validity.	
Parental Pain catastrophizing [50]	Pain Catastrophizing Scale – Parent report (PCS-P)	Baseline, 8 weeks, 16 weeks, 10 and 12 months
	Measures parental catastrophizing about their child’s pain.	
	13 items rated on a five-point scale (0–4). Total scores range 0–52 with higher scores indicating more catastrophic thinking by the parents.	
	Psychometric properties are good to very good.	
Adult response to children’s symptoms [54]	Adult response to children’s symptoms scale (ARCS)	Baseline, 8 weeks, 16 weeks, 10 and 12 months
	Parent self-report measure of a range of parental responses to pain of their child.	
	29 items rated on a five-point scale (Never-always). Consists of subscales 1) Protective responses, 2) minimizing responses (criticizing or downplaying the pain) and 3) monitoring/encouraging responses (encouraging activity while monitoring symptoms)	
	Valid instrument for assessing parents’ responses to children’s pain for diverse chronic pain symptoms.	
Other study parameters	Used in economic evaluation and process evaluation	
Costs (economic evaluation) [55]	Cost diary	After treatment each month until month 12.
	Health care utilization, school absence and productivity losses are recorded.	
Generic health related quality of life (economic evaluation) [56]	EQ-5D-Y	Baseline, 8 weeks, 16 weeks, 10 and 12 months
	Generic self-report measure of health related quality of life.	
	5 Items, scored on three levels: no problems, moderate problems, severe problems. Contains domains 1) mobility, 2) self-care, 3) usual activities, 4) pain/discomfort, and 5) anxiety/depression. 1 visual analogue scale to rate their own health between 0 and 100 (best health state).	
Satisfaction/patient centeredness (Process evaluation) [58]	Giving Youth A Voice Questionnaire (GYV-20)	16 weeks
	The instrument has 4 themes, 1) supportive and respectful relationships, 2) Information sharing and communication, 3) Supporting independence and 4) Teen-centred service. Each item is formulated as a question, starting with ‘How much do the people who work with you…’ and then a description of a specific action or behaviour of the health care professional is given. Response options range from 1–7, with a ‘not applicable’ category added. Scale scores can be calculated as the mean of the ratings for the items in the scale.	
Satisfaction/family centeredness (Process evaluation) [59, 60]	Measure of Processes Of Care – Parent form – Short form (MPOC-P-20)	16 weeks
	20 items, scored on a scale 1–7. Consists of 5 scales, 1) Enabling and Partnership, 2) Providing General Information, 3) Providing Specific Information about the Child, 4) Coordinated and Comprehensive Care for the Child and Family and 5) Respectful and Supportive Care. Responses to each item are converted to a mean for each scale. No total score can be calculated.	
Treatment expectations of children (Process evaluation) [61]	Credibility/Expectancy Questionnaire (CEQ-Adolescent)	Baseline
	On the 11 items (5 on credibility, 6 on expectancy) can be answered on a 9-point scale from ‘totally not’ to ‘totally’. Total scores are a sum score of the individual items and ranges from 11 to 99.	
Treatment expectations parents (Process evaluation) [61]	Credibility/Expectancy Questionnaire (CEQ-Parent)	Baseline
	On the 11 items (5 on credibility, 6 on expectancy) can be answered on a 9-point scale from ‘totally not’ to ‘totally’. Total scores are a sum score of the individual items and ranges from 11 to 99.	
Joint Hypermobility Syndrome (HMS) [29, 32, 43]	Birghton Criteria	Baseline
	To identify adolescents with HMS the Brighton criteria, including a Beighton score will be used. A cut-off value of ≥5 will be used for hypermobility.	

^a^Not for all measures detailed information about reliability and validity was available

#### Other study parameters

Demographic variables will be measured at baseline. These variables include age, sex, educational level and school absence, family composition, ethnic background. Variables related to chronic pain that will be measured include: onset and duration of pain complaints, location of pain complaints, course of the complaints, and (if applicable) medication use.

#### Costs measurement

To evaluate the economic consequences, the intervention costs, other health care costs, patient and family costs, and productivity losses will be assessed. Patient and family costs include out-of-pocket costs, such as costs for informal care and extra expenses. Productivity losses (for adolescents and parents) are based on the days absent from work or school because of the pain.

At baseline, total costs will be inventoried 3 months retrospective. During the trial period (12 months) monthly cost-diaries will be used after the end of treatment. For addressing cost-effectiveness of interventions, the cost-diary method might be a successful method [55]. Both the parents and the adolescents will be asked to provide information about health care utilization and school absenteeism/productivity losses. If the adolescents are 18 years or older, they may choose to fill out the cost diary themselves, below 18 years it is recommended they complete the cost diary together with their parents.

Generic health related quality of life was measured with the EQ-5D-Y [56]. The EQ-5D-Y is a self-report measure of health related quality of life. QALY’s can be calculated by means of the applying a tariff to obtain a weighted health state index. The adult tariff will be used for the Youth-version of the questionnaire, since no youth-tariff is available.

#### Process evaluation

To evaluate treatment fidelity, a process evaluation will be performed. The Method of Assessing Treatment Delivery (MATD) [57] will be applied, including audio or videotaping treatment sessions to assess protocol adherence. There will be a focus on protocol adherence of the therapists in the MRP condition and a contamination check will be performed to evaluate whether treatments provided in both arms of the trial were indeed different from each other. For treatment fidelity to be adequate, at least 70 % of essential treatment elements must have occurred in order to satisfy the requirements for protocol adherence. Furthermore, maximally 10 % of prohibited treatment elements may have occurred to satisfy the requirements for contamination. Furthermore, satisfaction with the received treatment and the degree of adolescent/family centeredness of the offered interventions will be measured with the Giving Youth a Voice Questionnaire (GYV-20) [58] and Measure of Processes of Care Parent Version short form (MPOC-P-20) [59, 60]. Finally, a comparison will be made between treatment expectation (measured with the Credibility/Expectancy Questionnaire, CEQ-m) [61].

### Data-analysis

#### Statistical analysis

All analysis will be performed on the basis of the “intention-to-treat principle”. Descriptive statistics for demographic and clinical characteristic for both groups (MRP and CAU) and the total group will be used. An inventory of missing data will be made and if necessary an imputation strategy will be chosen.

#### Effect evaluation

Differences after 8 and 16 weeks of treatment will be calculated (short term outcomes), as well as long term outcomes at 10 and 12 months. A linear mixed models approach for calculating differences between baseline and the final follow-up at 12 months will be used. This method used both fixed and random effects in the same analysis. It handles naturally unbalanced data as e.g. uneven spacing of repeated measures and allows analysing the relationship of predictor covariates with the dependent variable (FDI-score). It also accounts successfully for the observed pattern of dependences in the measurements. Appropriate covariates (for the primary outcome potential important imbalances in baseline variables) will be identified in a univariate regression analysis. Before starting the analysis the baseline score of the dependent variable FDI-score and all identified covariates will be centred by subtracting the group mean.

In a first step a fixed effects model will be run and in a second step random effects will be added. Insignificant covariates will then be stepwise removed from the model. Model fit will be assessed with the help of the Bayesian Information Criterion (BIC) and the -2Log likelihood [62].

#### Economic evaluation

In the economic evaluation costs and effects of the intervention condition (MRP) and the control condition (CAU) will be calculated and compared, using a societal perspective. Total costs will be estimated using a bottom-up approach, where information on each element of service use is multiplied by an appropriate standardized unit cost and summed to provide and overall total cost. For the cost valuation, standardized cost prizes will be used from the Dutch manual for cost analysis in health care research [63]. Productivity losses will be calculated based on the Human Capital Approach.

At baseline resource use prior to the study will be measured, to allow this use as a covariate in the analysis, which enables correction for possible cost differences at baseline [64]. Cost per patient year (= participant year) will be calculated.

Intended, both a cost-effectiveness analysis (CEA) and a cost-utility analysis (CUA) will be performed. For the CEA the cost-effectiveness ratio will be stated in terms of cost per 5 points of improvement on the Functional Disability Inventory (FDI). For the CUA the cost-utility ratio will be stated in terms of cost per Quality Adjusted Life Year (QALY) gained, as measured by the EQ5D-Y [65]. Bootstrap re-sampling techniques will be used to test for differences and uncertainty in cost and effects between MRP and CAU.

#### Process evaluation

To evaluate treatment fidelity, audio or video recordings will be analysed of treatment sessions of both interventions conditions [42]. From these recordings, a random sample will be taken for analysis. The recordings are rated for protocol adherence, contamination, and differentiation [42]. Ratings will be performed by 2 raters, independent of this trial and blind to the study hypotheses. To enable rating, a Treatment Fidelity Checklist is developed where raters can indicate whether a treatment-element took place or not [57]. The percentage of deviation from the allowed treatment-elements is compared between the intervention and control condition by logistic regression analysis.

The data from the Credibility/expectancy questionnaire and the measures of patient/family centeredness (GYV-20 and MPOC-20) of the provided treatment will be used to evaluate differences in credibility and expectancy at the start of the treatment, and treatment satisfaction at the end of treatment.

## Discussion

Research on treatment options for adolescents with CMP is still in its infancy, but multidisciplinary treatment for this patient group seems to be a promising treatment approach. A limited number of studies have been performed to study the effectiveness of multidisciplinary treatments on improving functional ability [66–70]. Although several pre- and post-treatment comparisons have been performed, to our knowledge only few studies have compared different treatment options with each other. Another important element in adolescent treatment is the participation of parents, again, with limited evidence available on the effectiveness of parental interventions [17, 22, 38].

There is an urgent need for more research. Therefore, a pragmatic multicentre randomized controlled trial (RCT) was established to compare a new multimodal rehabilitation program with care as usual. For this study a more pragmatic approach is chosen. Schwarts and Lellouch [71] use the term ‘pragmatic’ to describe studies designed to choose between options of care, as opposed to ‘explanatory’ studies that test causal research hypotheses. Our aim is to compare the new treatment approach to existing practice to determine whether or not the new approach can be added to the treatment options existing presently. This approach led to some design choices that were made to maximize the applicability of the results to usual care settings.

To include a wide range of participants eligibility criteria are focused on the inclusion of all adolescent participants that would normally receive an indication for rehabilitation treatment. This should lead to the inclusion of a heterogeneous group of participants, very similar to the patients that are seen in current clinical practice.

Adolescents aged 12–21 years old are eligible to participate in the trial. Paediatric rehabilitation services are in principle provided up to the age of 18 years old. Most studies on treatments for adolescents include participants up to the age of 18 years old. The range of 12–21 years was chosen according to Kaplan’s [72] definition of adolescent life stage, starting with early adolescence at 12 years old, ending of late adolescence at 21 years old. Furthermore, research has shown that up to the age of 21 years, brain functions are still developing [73]. Therefore, interpretation of cognitive and behavioural processes can still be sensitive to change and can best be seen in to context of adolescence instead of adulthood. Additionally, in the Netherlands only 20 % of the 15–20 year olds are part of the working population, so for the largest part of this age group, their daily social and physical activities are more in line with the school-attending adolescents than with the working adult population.

To our knowledge, 2B Active is one of the first studies performing an economic evaluation on outpatient treatment and follow up, focussing both on the cost of the intervention itself and on patient and family costs. No measurement instrument existed that measured both adolescent and parent costs of the rehabilitation trajectory and follow-up. Therefore, a cost diary was developed to measure both medical consumption and productivity losses (work and school absenteeism) after completion of the rehabilitation program.

To summarize, several design choices that have been made may influence the balance between internal validity and external validity of the study. By studying the new intervention in a pragmatic way and in the setting in which the MRP treatment is intended to be offered, the results will be more generalizable to daily practice of rehabilitation care. Including a heterogeneous group of patients and analysis according to ITT may lead to results that are not over-estimated since real practice daily difficulties are incorporated in the trial results.

## Abbreviations

ARCS, adult responses to children’s symptoms scale; CAU, care as usual; CDI, child depression inventory; CEA, cost-effectiveness analysis; CEQ, credibility/expectancy questionnaire; CMP, chronic musculoskeletal pain; CUA, cost-utility analysis; FDI, functional disability inventory; FDI-P, functional disability inventory-parent report; FOPQ-C, fear of pain questionnaire- child version; FOPQ-P, fear of pain questionnaire-parent report; GA, graded activity; GE, graded exposure; GYV-20, giving youth a voice questionnaire; HMS, hypermobility syndrome; MATD, method of assessing treatment delivery; MPOC-P-20, measure of processes of care parent version short form; MRP, Multimodal Rehabilitation Program; PCS-C, pain catastrophizing scale – child version; PCS-P, pain catastrophizing scale-parent version; PHODA-Youth, photograph series of daily activities for youth; QALY, quality adjusted life year; QLA-CP, quality of life in adolescents with chronic pain; RCT, randomized controlled trial; VAS, visual analogue scale
